# Pulmonary Rehabilitation Programs: We Do Not Have All the Answers Yet

**DOI:** 10.3390/jcm14144993

**Published:** 2025-07-15

**Authors:** Vinicius Cavalheri, Kylie Hill

**Affiliations:** 1 Curtin School of Allied Health, Faculty of Health Sciences, Curtin University, Perth, WA 6102, Australia; k.hill@curtin.edu.au; 2 Allied Health, South Metropolitan Health Service, Perth, WA 6150, Australia

## 1. Introduction

Pulmonary rehabilitation has long been recognized as an important component of the care of people with chronic respiratory conditions [1,2]. These programs typically include exercise training, education, and the optimization of self-management and aim to address both pulmonary and extrapulmonary features of chronic respiratory disease [1]. The evidence supporting its efficacy to improve exercise capacity and health-related quality of life (HRQoL), and reduce dyspnoea, fatigue, and anxiety and depression, has largely been developed in people with chronic obstructive pulmonary disease (COPD) [3,4]. However, over the years, the evidence of its beneficial effects has been expanding to those with interstitial lung diseases (ILDs), asthma, pulmonary hypertension, bronchiectasis, and lung cancer, those recovering from COVID-19, and other populations [5].

Despite the known benefits of pulmonary rehabilitation, challenges to implementing these programs remain. Challenges include, but are not limited to, diverse funding models, poor referral to and uptake of pulmonary rehabilitation, the unavailability or scarcity of programs in certain areas/regions or for certain populations, and fixed structures to deliver pulmonary rehabilitation, which can prevent access to novel delivery models (e.g., telerehabilitation or home-based rehabilitation) [6]. There are also clinical questions that remain unanswered, such as the value of add-on interventions to pulmonary rehabilitation, focused on providing benefits in terms of specific health-related outcomes beyond the benefits of pulmonary rehabilitation alone.

The studies published in this Special Issue of the *Journal of Clinical Medicine* address some of these challenges and unanswered questions, offering fresh insights and approaches to pulmonary rehabilitation.

## 2. An Overview of Published Articles

The series of six recent studies [7,8,9,10,11,12] published in this Special Issue of the *Journal of Clinical Medicine* offer substantial contributions to the field of pulmonary rehabilitation.

The study by Klimczac et al. [7] explored changes following a much briefer, but more intense pulmonary rehabilitation program (PRP) than is usually offered. That is, most PRPs offer an eight-week program with twice weekly supervised exercise training (approximately 16 sessions). In contrast, Klimczak et al. offered daily exercise sessions for three weeks (15 sessions). At the end of this 3-week program, compared with measures collected prior to the program, gains were seen in the usual outcome measures such as health status and exercise tolerance. Although these changes are encouraging, without a control group, it is difficult to attribute all change to the intervention itself. Nevertheless, this study provides some support for shorter, more intense programs, which might be required to meet funding requirements from certain insurance companies and/or national health funds.

The randomized controlled trial (RCT) conducted by Williams et al. [8] addressed the question regarding the benefit of a specific add-on intervention to a PRP, cognitive behavioral therapy (CBT), which was designed to specifically address the sensation of breathlessness in people with COPD. The CBT program was run by a clinical psychologist, parallel to the standard eight-week pulmonary rehabilitation program. Each of the eight CBT modules included education, individual reflection, and tasks/homework activities focused on managing breathlessness. The comparison group attended a weekly one-hour social group session to balance the time spent by participants undergoing CBT. A total of 101 participants were included, and findings suggested that the inclusion of CBT did not provide clear additional benefits in terms of feelings of anxiety, depression or six-minute walk distance at 1, 6, or 12 months post-intervention beyond those achieved by adding a social group. This may be because, at least in part, the exercise training component of a PRP, along with reassurance from a physiotherapist, serve as graded behavior experiments, which are a powerful way help the person develop a new nonthreatening understanding of their dyspnoea [13]. Because PRPs are such an effective intervention, studies which explore the value of an adjunct or ‘add-on’ therapy are often unable to demonstrate additional benefits [14].

There were two studies that focused specifically on the exercise component of pulmonary rehabilitation in people with lung cancer [9,10]. Edbrooke et al. [9] conducted a review of systematic reviews and aimed to summarize the effectiveness of exercise training across the lung cancer care continuum (i.e., pre- and post-operatively and in people with inoperable disease). Whish-Wilson et al. [10] investigated, via a survey, current exercise practices in Australia and New Zealand for people undergoing surgical resection for lung cancer. The review by Edbrooke at al. [9] included 30 systematic reviews, mostly in people with operable lung cancer (n = 28). The findings suggested that (i) pre-operative exercise reduces post-operative pulmonary complications and improves exercise capacity; (ii) post-operative exercise improves exercise capacity and muscle strength; and (iii) meta-analyses of exercise interventions in people with inoperable lung cancer demonstrated inconsistent findings. The effect on health-related quality of life was unclear. Of note, despite the emerging evidence for the benefits of pre- and post-operative exercise training in people with lung cancer, the survey by Whish-Wilson et al. [10] demonstrated that the availability and uptake of pre- and post-operative exercise training in Australia and New Zealand remain low. The survey had a total of 70 respondents from services across the two countries and reported that pre-operative exercise programs were provided by only eight (11%) services and that half of the respondents referred less than 25% of patients to post-operative exercise programs on hospital discharge. The authors concluded that exercise programs for people undergoing surgical resection for lung cancer are still scarcely available across Australia and New Zealand and referral to existing programs is still not routine practice. This may show, at least in part, that PRPs are a finite resource which tend to be reserved for people with progressive chronic lung diseases, such as chronic obstructive pulmonary disease, for which there is overwhelming evidence of benefit.

As a response to the COVID-19 pandemic, pulmonary rehabilitation delivered via telehealth has emerged as a promising solution for both people recovering from COVID-19 and people with chronic respiratory disease in general. Pescaru et al. [11] conducted a systematic review to explore studies that reported on pulmonary rehabilitation delivered via telehealth in people recovering from COVID-19. Seven studies (a total of 412 participants), with diverse methods, were included. Three of these were randomised trials and no meta-analyses of the data were attempted. The length of the telerehabilitation programs ranged between 4 and 10 weeks and the programs were delivered via a mobile app or video call. Exercise training programs included aerobic and resistance training, breathing exercises, and functional activities. The findings were generally positive for one or more of the pre-specified outcomes (physical health, mental health, health-related quality of life and dyspnoea). Further work is needed to understand the optimal training approach for this clinical population.

Lastly, Krinski et al. [12] carefully characterized those with interstitial lung disease who met the criteria to receive end-of-life care (n = 14) with those who did not (n = 44). Despite similarities between the groups in terms of age, the proportion of females, and type of pharmacotherapy, those in the end-of-life care group had a greater proportion of people with idiopathic pulmonary fibrosis, co-morbid conditions, and lower functional status, measured as exercise tolerance and muscle force. They were also higher uses of healthcare in terms of annual hospitalizations. These data highlight the importance of promoting physical activity in this population as a means to reduce caregiver burden.

## 3. Conclusions and Future Directions

The studies presented in this Special Issue underscore the widespread applicability of pulmonary rehabilitation beyond COPD, including asthma, lung cancer, post-COVID-19 recovery, as well as the potential role of pulmonary rehabilitation teams in contributing to assessment and management plans for people with ILD who meet the criteria for end-of-life care.

The future of pulmonary rehabilitation seems promising, with several potential areas available for further development. While the evidence supporting the benefits of pulmonary rehabilitation in people with chronic respiratory diseases continues to grow, challenges in funding models, accessibility, and the integration of flexible, personalized and novel models of pulmonary rehabilitation, amongst others, persist. Addressing these barriers is crucial to maximizing the reach and impact of pulmonary rehabilitation programs across diverse patient populations.
